# Microwave Irradiation: Alternative Heating Process for the Synthesis of Biologically Applicable Chromones, Quinolones, and Their Precursors

**DOI:** 10.3390/molecules26206293

**Published:** 2021-10-18

**Authors:** Hélio M. T. Albuquerque, Diana C. G. A. Pinto, Artur M. S. Silva

**Affiliations:** LAQV-REQUIMTE, Department of Chemistry, University of Aveiro, 3810-193 Aveiro, Portugal; helio.albuquerque@ua.pt (H.M.T.A.); diana@ua.pt (D.C.G.A.P.)

**Keywords:** microwave irradiation, organic synthesis, heterocyclic compounds, (*E*/*Z*)-3-styrylchromones, (*E*)-1-[3-(2-hydroxyphenyl)-4-styryl-1*H*-pyrazol-1-yl]ethan-1-ones, (*E*)-2-(4-arylbut-1-en-3-yn-1-yl)chromone

## Abstract

Microwave irradiation has become a popular heating technique in organic synthesis, mainly due to its short reaction times, solventless reactions, and, sometimes, higher yields. Additionally, microwave irradiation lowers energy consumption and, consequently, is ideal for optimization processes. Moreover, there is evidence that microwave irradiation can improve the regioselectivity and stereoselectivity aspects of vital importance in synthesizing bioactive compounds. These crucial features of microwave irradiation contribute to its inclusion in green chemistry procedures. Since 2003, the use of microwave-assisted organic synthesis has become common in our laboratory, making our group one of the first Portuguese research groups to implement this heating source in organic synthesis. Our achievements in the transformation of heterocyclic compounds, such as (*E*/*Z*)-3-styryl-4*H*-chromen-4-ones, (*E*)-3-(2-hydroxyphenyl)-4-styryl-1*H*-pyrazole, (*E*)-2-(4-arylbut-1-en-3-yn-1-yl)-4*H*-chromen-4-ones, or (*E*)-2-[2-(5-aryl-2-methyl-2*H*-1,2,3-triazol-4-yl)vinyl]-4*H*-chromen-4-ones, will be discussed in this review, highlighting the benefits of microwave irradiation use in organic synthesis.

## 1. Introduction

**Figure 1:**
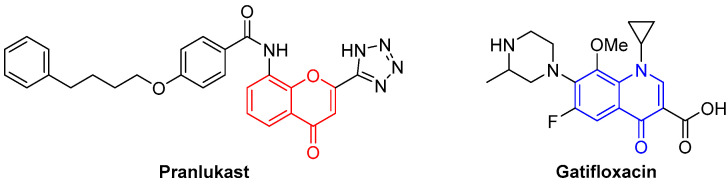
Examples of chromone- and quinolone-containing drugs.

Organic synthesis, essential in producing new biologically active compounds, particularly for potential pharmaceutical use, is commonly associated with dangerous and toxic procedures, especially for the environment. As so, scientists are more and more focused on developing innovative environmentally friendly methodologies, following as much as possible the Green Chemistry Principles (GCP) [1,2]. In this context, although not consensual, microwave-assisted organic synthesis seems to be an excellent approach to fulfill some of the GCP [3,4,5]. The design for energy efficiency seems obvious, but safer solvents and auxiliaries and fewer derivatives’ production are also achieved principles when microwave irradiation is employed. There are some shreds of evidence that microwave irradiation heating is achieved through the interaction of electromagnetic radiation at the molecular level, thereby producing the necessary energy to accomplish the reaction [6,7]. Additionally, it was established that in some reactions the electromagnetic wave effect is involved in the electron transfer process [8]. Moreover, there is evidence that microwave irradiation use is very efficient in synthesizing heterocyclic compounds, making it an essential heating source in developing new potential drugs [9]. One of the main focuses of our research group is the synthesis of both oxygen and nitrogen heterocyclic compounds, being the foremost examples of this kind of heterocycles, chromone, and quinolone derivatives. These heterocycles are often found in market drugs such as pranlukast and gatifloxacin (Figure 1), used to treat allergic rhinitis and as antibacterial agents, respectively [10,11].

Given that microwave irradiation was established in our research group, it is now a common energy source in our daily laboratory work, aiming to obtain new bioactive compounds in fewer minutes, using less amounts of solvents, and getting fewer by-products. In this review, we discuss the most noticeable features obtained in some common transformations by using microwave irradiation, highlighting the type of heterocyclic compounds obtained and, when possible, the effect of microwave irradiation on the transformation stereochemistry.

## 2. Microwave-Assisted Aldol Condensation in the Synthesis of Chalcones

**Scheme 1:**
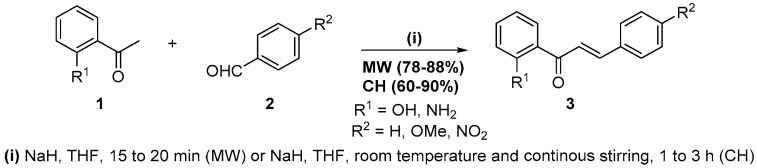
Synthesis of chalcones using a Milestone-MicroSynth.

The aldol condensation is one of the most valuable strategies to form carbon-carbon bonds, apparently was first introduced in 1872 by Charles Wurtz, and consists of the condensation of two carbonyl compounds (ketones or aldehyde) in the presence of an acid or a base as a catalyst [12,13]. This condensation is of particular application in the synthesis of chalcones, recognized bioactive flavonoids [14] and important precursors of chromones and quinolones. Our experience in their synthesis [15,16] indicates that the best results are obtained with base catalysis and usually a long reaction time (3 h to 20 h). Therefore, aiming to shorten the reaction time, we tested the aldol condensation of 2′-hydroxyacetophenones **1** with benzaldehydes **2** under microwave irradiation (Scheme 1) [17].

We should highlight a few points concerning the methodology. Obviously, the achieved objective was shortening the reaction time to 15 min when 2′-hydroxyacetophenone was used and to 20 min for the 2′-aminoacetophenone. The yields were excellent for both electron-donating and -withdrawn groups, and the procedure is straightforward: We can apply it in an undergraduate organic synthesis laboratory. Furthermore, it is possible to scale up to 500 g if it is used as a Milestone-MicroSynth with several vessels.

## 3. Microwave-Induced Baker-Venkataraman Rearrangement for the Synthesis of Chromones

**Scheme 2:**
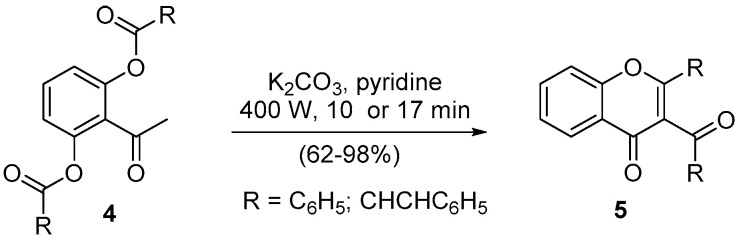
Synthesis of 3-aroyl- and 3-cinnamoyl-4*H*-chromen-4-ones using a Milestone-MicroSynth.

Chromones (4*H*-chromen-4-ones) and the corresponding nitrogen analogues, quinolones (quinolin-4(1*H*)-one), are recognized for their vast biological activity [11,18], a fact that also encourages their synthesis. The Baker–Venkataraman rearrangement is one of the oldest methodologies to obtain chromone derivatives [19], and we established some modifications that simplify the procedures [20,21]. However, in classical heating conditions, the reaction time is always long, 1 or more hours, and sometimes more than one product is obtained [21]. Therefore, the use of microwave irradiation to perform the transformation seemed to be a good approach to shortening the reaction time and simultaneously using a greener energy source. In that regard, both 3-aroyl-2-aryl-4*H*-chromen-4-ones (**5**, R = C_6_H_5_) and 3-cinnamoyl-2-[(*E*)-styryl]-4*H*-chromen-4-ones (**5**, R = CHCHC_6_H_5_) were obtained with yields above 60% (Scheme 2) [22,23]. The main achievement of these transformations using microwave irradiation was the reduced reaction time and the scale-up to nearly 1 kg, using several vessels simultaneously. Still, the fact that the reaction does not need a nitrogen atmosphere is also important because it reduces its cost.

**Scheme 3:**
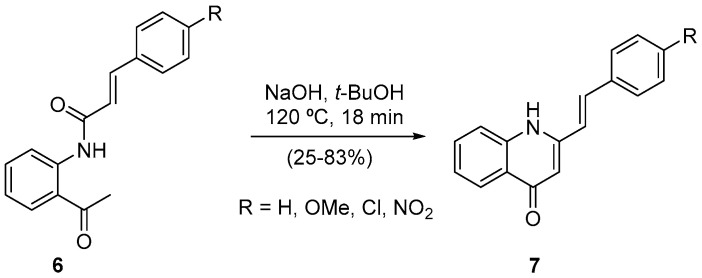
Synthesis of (*E*)-2-styrylquinolin-4(1*H*)-ones using a Milestone-MicroSynth.

A similar procedure was used to shorten the reaction time of the (*E*)-2-styrylquinolin-4(1*H*)-ones **7** synthesis through a one-pot process involving the cinnamoyl transposition and cyclization (Scheme 3) [24]. We achieved the desired reduced reaction time, and the developed methodology, under microwave irradiation, which had two extra positive effects: the use of a less dangerous base and the possibility to obtain, although in lower yield (25%), the (*E*)-2-(4-nitrostyryl)quinolin-4(1*H*)-one, which is not obtained in classical heating conditions [24].

## 4. Microwave-Assisted Knoevenagel Condensation in 3-Styryl-4*H*-chromen-4-ones Synthesis

**Scheme 4:**
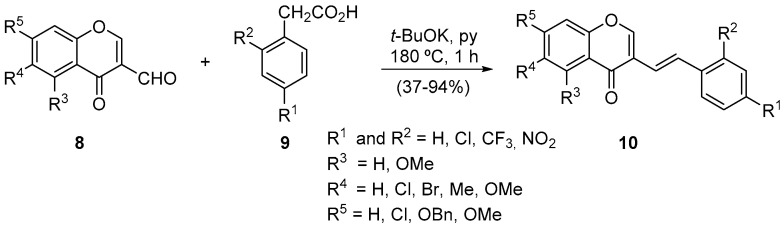
Synthesis of (*E*)-3-styryl-4*H*-chromen-4-ones using a Milestone-MicroSynth and a CEM model Discover SP.

Knoevenagel condensation, a reaction named after being established by the German chemist Emil Knoevenagel [25,26,27], is commonly used in organic synthesis and is of particular utility in total synthesis [28]. In our case, we applied this type of reaction to obtain chromone derivatives, in particular (*E*)-3-styryl-4*H*-chromen-4-ones **10** [29]. The methodology consists of the Knoevenagel condensation between the 4*H*-chromen-4-one-3-carbaldehyde **8** with arylacetic acids **9**, followed by an *in situ* decarboxylation (Scheme 4).

The use of microwave irradiation profit was the massive cut in the reaction time, from 12 h to 31 h in classical heating conditions to 1 h under microwave conditions [29]. Moreover, it was observed that the presence of a nitro group in the *para* position of the arylacetic acid **9** lowers the yield of the corresponding (*E*)-3-styryl-4*H*-chromen-4-ones **10**, both in classic (48%) and microwave conditions (56%), with the microwave improving the yield slightly. Later on, we applied the methodology successfully to different 4*H*-chromen-4-one-3-carbaldehyde derivatives **8**. Although the (*E*)-5-methoxy-3-styryl-4*H*-chromen-4-one obtained 37%, the method seems to be efficient and, above all, diastereoselective to prepare the (*E*)-isomer (Scheme 4) [30].

4*H*-Chromen-4-one-3-carbaldehydes condensation with phenylmalonic acid was also performed under microwave irradiation (120 W) at 100 °C for 7 min [31]. The yields were slightly lower (from 47% to 61%), and the diastereoselectivity towards (*E*)-isomer was maintained. Additionally, the reaction was performed in solid support, the sodium acetate, adding an additional greener effect to this procedure, eliminating a solvent.

## 5. Microwave-Induced Heck Reaction towards the Synthesis of (*E*)-3-styrylflavones and (*E*)-3-styrylquinolin-4(1*H*)-ones

In 1972, Heck and Nolley reported a halide-olefinic substitution catalyzed by palladium catalysis [32]. Since then, not only the reaction is generally called Heck reaction but also proved to be suitable in the synthesis of several organic compounds [33,34], including natural products [35] and more complex molecules [36].

**Scheme 5:**
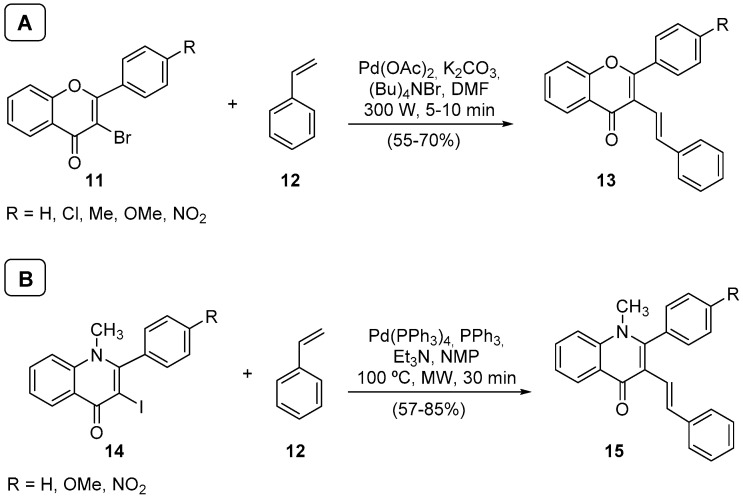
(**A**) Synthesis of (*E*)-3-styrylflavones; (**B**) synthesis of (*E*)-3-styrylquinolin-4(1*H*)-ones using a Milestone-MicroSynth.

Knowing that microwave irradiation is well suited for Heck reaction [37], promoting maximal interaction between reagents and minimizing the formation of undesired side products, we conceived a program aiming to obtain (*E*)-3-styrylflavones **13** and (*E*)-3-styrylquinolin-4(1*H*)-ones **15** from 3-bromoflavones **11** and 2-aryl-3-iodo-1-methylquinolin-4(1*H*)-ones **14**, respectively (Scheme 5) [38,39].

The 3-Bromoflavones **11**, although suitable substrates for Heck reaction with styrene derivatives, have some steric hindrance, which can explain the lower yields obtained in the experiments performed at classical heating conditions. Such disadvantages could be overcome using microwave irradiation as a heating source. The yields were much better and the reaction was also accomplished in less than 10 min (Scheme 5A). Additionally, the presence of electron-withdrawing or -donating groups in the flavone B-ring did not prevent the transformation, and some interesting derivatives could also be obtained [39]. Another important feature of the developed methodology is its stereoselectivity. The obtained (*E*)-3-styrylflavones **13** presents the (*E*)-configuration of the vinylic system and the s-*cis* stereochemistry, as depicted in structure **13**. NMR data can confirm both configurations, particularly the vinylic system coupling constant *J* = 17 Hz, typical of an (*E*)-configuration and the high chemical shift of the β-proton due to the anisotropic carbonyl deshielding [38].

We also extended our interest in this transformation to the use of 2-aryl-3-iodo-1-methylquinolin-4(1*H*)-ones **14**, which allowed the synthesis of (*E*)-3-styrylquinolin-4(1*H*)-ones (**15**) in good yields (Scheme 5B) [39]. Naturally, the reaction conditions needed to be revised to these derivatives, mostly due to the 2-aryl-3-iodo-1-methylquinolin-4(1*H*)-ones **14** solubility and reactivity; but, overall, the results were excellent. We can highlight that the reaction time was short, and the product was again obtained with high stereoselectivity.

## 6. Diels–Alder Reactions Using 4*H*-chromen-4-ones and Quinolin-4(1*H*)-ones as Dienes

**Scheme 6:**
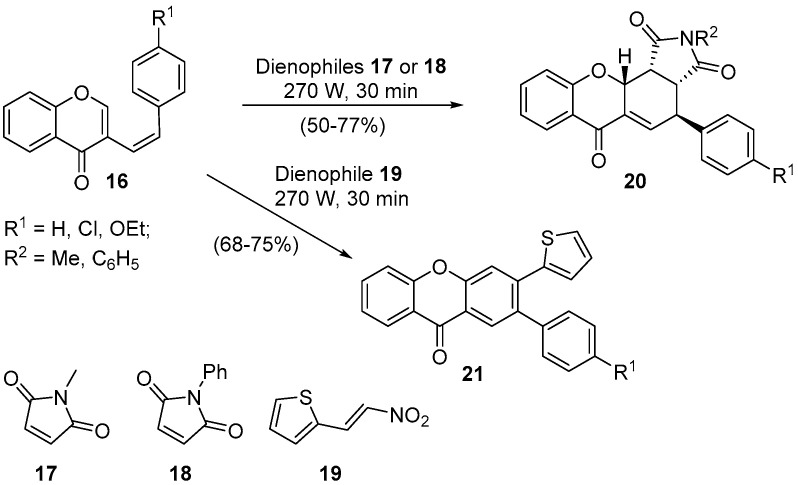
Solvent-free synthesis of *endo*-4-aryl-2-methyl-3a,4,11a,11b-tetrahydrochromeno[2,3-*e*]isoindole-1,3,6(2*H*)-triones **20** and 2-aryl-3-(thiophen-2-yl)-9*H*-xanthen-9-ones **21** using a Prolabo MX350 and a Milestone-MicroSynth.

In 1928 Otto Diels and his student Kurt Alder published their first paper concerning a cycloaddition reaction that henceforth is known as the Diels–Alder (DA) reaction [40], among the most significant and adaptable reactions known in organic synthesis [41,42,43,44]. Notably, we initiated our interest in MW-assisted organic synthesis with a DA-reaction, delivering in 2003 the first literature report of MW-assisted DA reaction using 3-styryl-quinolin-4(1*H*)-ones as substrates [45]. Since then, our research group has kept its interest in developing MW-assisted methods for synthesizing a diversity of compounds based on both inter- and intramolecular DA reactions.

(*Z*)-3-Styryl-4*H*-chromen-4-ones **16**, readily available through a Wittig reaction between 4*H*-chromen-4-one-3-carboxaldehydes and benzylidenetriphenylphosphoranes [46], proved to be a low-reactive diene under classical heating conditions, even when a very reactive dienophile, such as *N*-methylmaleimide, was employed [47]. Mixtures of compounds were obtained and the desired adduct, 4-aryl-2-methyl-3a,4,11a,11b-tetrahydrochromeno[2,3-*e*]isoindole-1,3,6(2*H*)-trione, was obtained in yields below 50%. Aiming to establish a more efficient synthetic procedure, we started our interest in the use of MW irradiation to improve the (*Z*)-3-styryl-4*H*-chromen-4-ones **16** reactivity in the DA reactions (Scheme 6) [45,47].

The first point to be mentioned is the fact that the DA reaction of electron-deficient dienophiles *N*-methylmaleimide **17**, *N*-phenylmaleimide **18**, and 2-(2-nitrovinyl)thiophene **19** with (*Z*)-3-styryl-4*H*-chromen-4-ones **16** produces the respective cycloadducts in very good yields, in solventless conditions, and in 30-min reaction time (Scheme 6) [45,47]. It should also be highlighted that in the reaction with 2-(2-nitrovinyl)thiophene **19**, the DA adduct undergoes *in situ* HNO_2_ elimination and oxidation, giving 2-aryl-3-(thiophen-2-yl)-9*H*-xanthen-9-ones **21** regioselectively towards the depicted isomer **20** (Scheme 6).

**Scheme 7:**
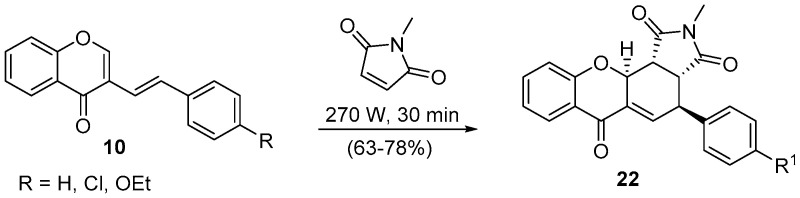
Solvent-free synthesis of *exo*-4-aryl-2-methyl-3a,4,11a,11b-tetrahydrochromeno[2,3-*e*]isoindole-1,3,6(2*H*)-triones using a Prolabo MX350 and a Milestone-MicroSynth.

Considering the reaction with the dienophiles **17** and **18**, besides the good reaction yields, it is noteworthy the stereoselectivity of the reaction towards the *endo* cycloadduct is depicted in structure **20** (Scheme 6). This result was also supported by ab initio calculations, which showed that the thermodynamically controlled product was formed [47]. Remarkably, the *exo* cycloadduct **22** can be obtained in the DA reaction using (*E*)-3-styryl-4*H*-chromen-4-ones **10** as dienes (Scheme 7) [45,47].

**Scheme 8:**
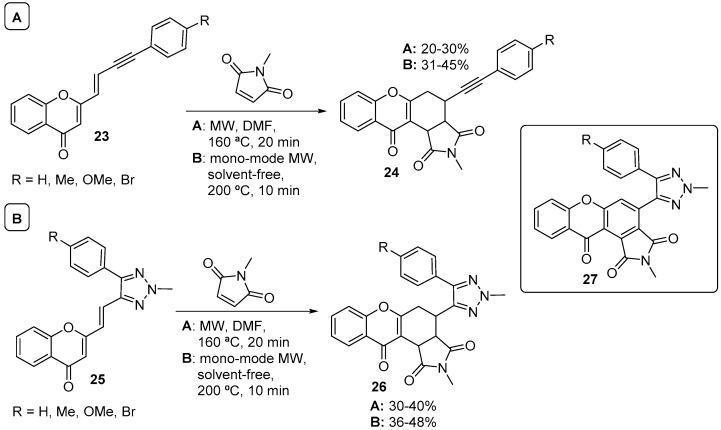
Synthesis of 9*H*-xanthen-9-one-1,2,3-triazole hybrids **27** via cycloadducts **24** (**A**) and **26** (**B**) using a Milestone-MicroSynth and a CEM model Discover SP.

Following these earlier studies in DA reactions with (*E*)- and (*Z*)-3-styryl-4*H*-chromen-4-ones as dienes, years later, we also explored the reactivity of particular chromone derivatives **23** and **25** to develop a method for the synthesis of 9*H*-xanthen-9-one-1,2,3-triazole hybrids **27** (Scheme 8) [48]. The diene of chromones **23** showed very low reactivity in classical heating conditions using maleimides as dienophiles. Fortunately, when multimode MW irradiation was applied, cycloadducts **24** were obtained in 20–30% yield in 20-min reaction time (Scheme 8A). Using a monomode microwave apparatus, we further improved the reaction yield to 31–45%, shortening the reaction time from 20 min to 10 min and avoiding the use of solvents (Scheme 8). The same principle was applied to chromone derivatives **25** (1,2,3-triazole replacing the internal alkyne of chromones **23**), and again the best yields of cycloadducts **26** (36–48%) were achieved after 10 min of monomode MW irradiation, in solvent-free conditions (Scheme 8B).

**Scheme 9:**
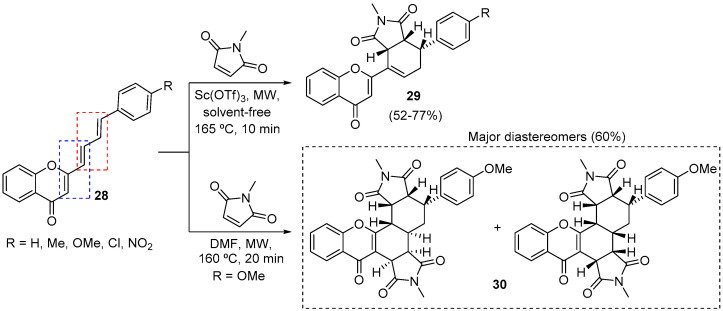
MW-assisted synthesis of monoadducts **29** and bisadduct **30** using a Milestone-MicroSynth and a CEM model Discover SP.

Following these studies, we decided to replace the internal alkyne of chromones **23** for an additional double bond to came up with 4*H*-chromen-4-one compounds with two dienes **28** (Scheme 9) [49]. This particular type of compound has two possible DA reaction sites, the reactivity of which was further explored using MW irradiation. If Sc(OTf)_3_ is used as Lewis acid in the solvent-free DA reaction of 4*H*-chromen-4-ones **28** with *N*-methylmaleimide, monoadducts **29** are obtained in 52–77% yield (Scheme 9). On the other hand, in the absence of this Lewis acid, it was possible to drive the DA reaction towards the bisadduct **30** in 60% yield, obtained as a mixture of two diastereomers (Scheme 9).

**Scheme 10:**
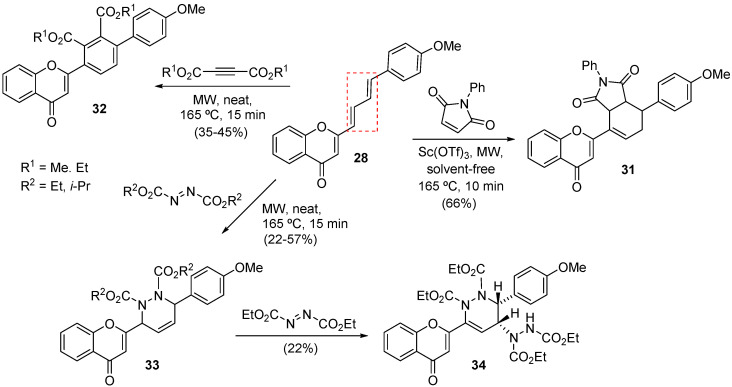
Solvent-free synthesis of compounds **31**, **32**, **33**, and **34** using a CEM model Discover SP.

The MW-assisted DA reaction scope was extended further to other dienophiles such as *N*-phenylmaleimide, acetylenedicarboxylates, and azodicarboxylates, which gave the compounds **31**, **32**, and **33** in 66%, 35–45%, and 22–57% yield, respectively (Scheme 10) [49]. MW irradiation also promoted a subsequent Alder-ene reaction involving compound **33** and diethyl azodicarboxylate (DEAD), which synthesized compound **34** in a 22% yield (Scheme 10).

**Scheme 11:**
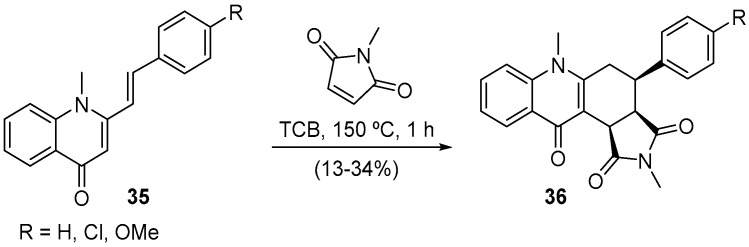
Synthesis of 4-aryl-2,6-dimethyl-4,5,6,11b-tetrahydro-1*H*-pyrrolo[3,4-*a*]acridine-1,3,11(2*H*,3a*H*)-triones using a Milestone-MicroSynth and a CEM model Discover SP.

Given the Diels–Alder reaction using 3-styryl-4*H*-chromen-4-ones **10** and **16** as dienes success, we tested less reactive derivatives, the (*E*)-1-methyl-2-styrylquinolin-4(1*H*)-ones **35** in the same transformation (Scheme 11) [50]. The microwave irradiation effect was mostly observed in shortening the reaction time (from 72 h to 1 h) and restraining the formation of other products. Interesting is the fact that the expected cycloadduct, the 4-aryl-2,6-dimethyl-4,6,11a,11b-tetrahydro-1*H*-pyrrolo[3,4-*a*]acridine-1,3,11(2*H*,3a*H*)-triones, underwent *in situ* a 1,3-hydrogen shift, that is, the proton H-11a was shifted to carbon C-5, giving the final product **36** (Scheme 11) [50].

**Scheme 12:**
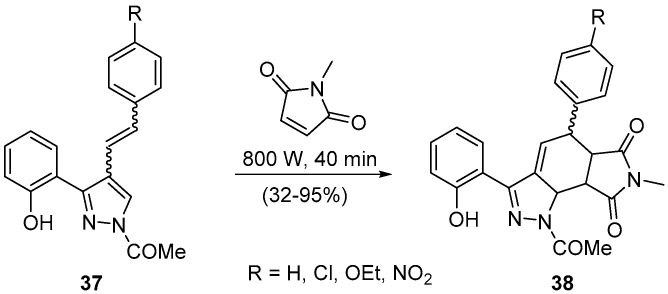
Solvent-free synthesis of 1-acetyl-5-aryl-3-(2-hydroxyphenyl)-7-methyl-5,5a,8a,8b-tetrahydropyrrolo[3,4-*g*]indazole-6,8(1*H*,7*H*)-diones using a Milestone-MicroSynth.

Holding in mind our interest in nitrogen heterocycles and the fact that we had, from another project, (*E*)- and (*Z*)-3-(2-hydroxyphenyl)-4-styryl-1*H*-pyrazoles [51,52], we decided to study their reactivity as dienes in DA reactions induced by microwave irradiation [53,54]. First, we would like to emphasize that it was a requisite of the *N*-derivatization to avoid other transformations [53]; so we used in the DA reaction the (*E*)- and (*Z*)-1-(3-(2-hydroxyphenyl)-4-styryl-1*H*-pyrazol-1-yl)ethan-1-ones **37** (Scheme 12) [53,54].

Besides the transformation achievement, good overall yields and short reaction time ought to be accentuated for some stereochemical facts. The DA transformation is stereoselective, giving the *endo* cycloadducts, 1-acetyl-5-aryl-3-(2-hydroxyphenyl)-7-methyl-5,5a,8a,8b-tetrahydropyrrolo[3,4-*g*]indazole-6,8(1*H*,7*H*)-diones **38**. Significant is the different 5-aryl orientations obtained for each isomer. That is, for (*E*)-isomer **37**, the C-5 of the cycloadduct **38** presented a (S*)-configuration, whereas for the (*Z*)-isomer **38** it was (R*) [53,54]. It was also evident that the (*Z*)-isomer **37** was less reactive due to the steric hindrance caused by the β-aryl group (Scheme 12).

**Scheme 13:**
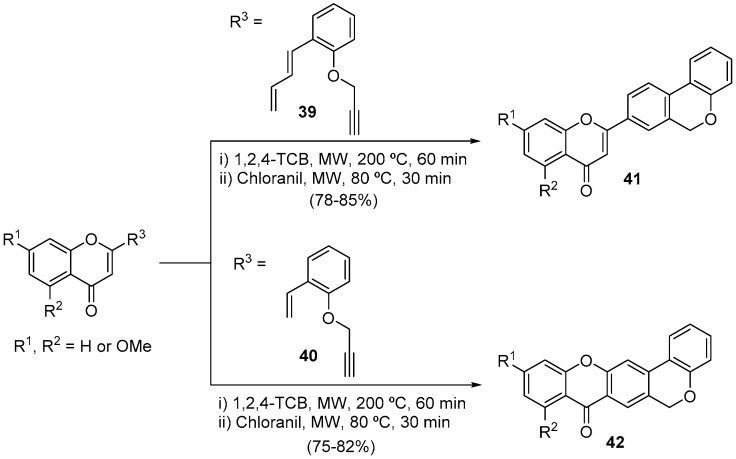
Synthesis of 2-(benzo[*c*]chromenyl)-4*H*-chromen-4-ones and chromeno[3,4-*b*]-9*H*-xanthen-9-ones using a CEM model Discover SP.

The interest of our research group in MW-assisted DA reactions for the synthesis of heterocycles dates back to 2003. However, the intramolecular version of the DA reaction using 4*H*-chromen-4-one derivatives was only reported 15 years later [55], with the synthesis of 2-(benzo[*c*]chromenyl)-4*H*-chromen-4-ones **41** and chromeno[3,4-*b*]-9*H*-xanthen-9-ones **42** in 78–85% and 75–82% yield, respectively, through intramolecular DA reaction followed by chloranil-mediated aromatization of *O*-propargyl 4*H*-chromen-4-ones with distinct unsaturated π-systems **39** and **40** (Scheme 13) [55].

**Figure 2:**
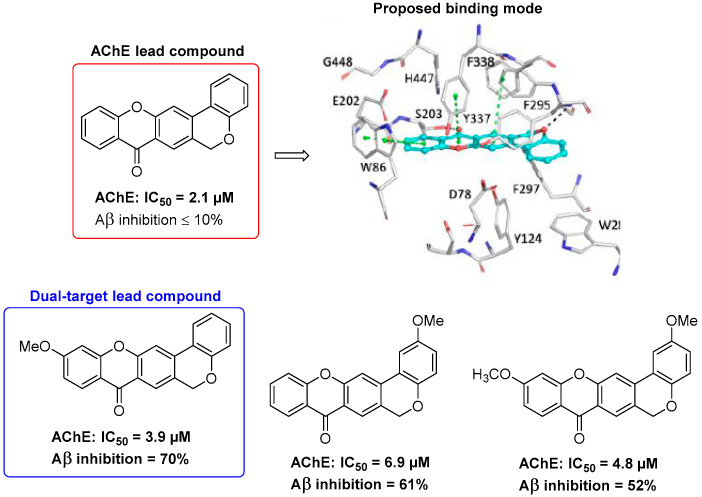
AChE inhibition, AChE binding mode, and Aβ aggregation inhibition of chromeno[3,4-*b*]-9*H*-xanthen-9-ones.

This intramolecular DA methodology allowed the efficient synthesis of a family of chromeno[3,4-*b*]-9*H*-xanthen-9-ones, a very interesting scaffold to develop multi-target drugs for Alzheimer disease [56]. This type of compound has a hybrid structure featuring the 9*H*-xanthen-9-one and chromene pharmacophoric units, with demonstrated anticholinesterase potency in the low-micromolar range (2.1 to 6.9 μM) and the ability to inhibit the amyloid-β (Aβ) 1–42 self-aggregation up to 70% (Figure 2). Docking studies allowed us to propose a binding mode for chromeno[3,4-*b*]-9*H*-xanthen-9-ones against acetylcholinesterase (AChE), grounded on the role of Ser203 and Phe294, which formed hydrogen-bonding interactions with the heterocyclic rings. The chromeno[3,4-*b*]-9*H*-xanthen-9-ones binding was stabilized by several π-stacking interactions between the main scaffold and aromatic residues in the binding pocket Trp86, Trp286, Phe338, and Tyr341. The slight differences in the binding affinity were probably due to the cost of the methoxy accommodation, resulting in small re-adjustments of the residues of the binding pocket. This explains why the unsubstituted derivative, which perfectly formed three π-stacking interactions and two hydrogen bonds, was the best compound exploited among all (Figure 2). Both experimental and computational insights allowed us to develop two lead compounds, depending on if the final goal was a single-target or a dual-target compound (Figure 2). Furthermore, we would like to highlight that chromeno[3,4-*b*]-9*H*-xanthen-9-ones are first-in-class AChE and Aβ aggregation dual inhibitors [56].

## 7. Tandem Reactions towards the Synthesis of Oxygen Polycyclic Compounds

**Scheme 14:**
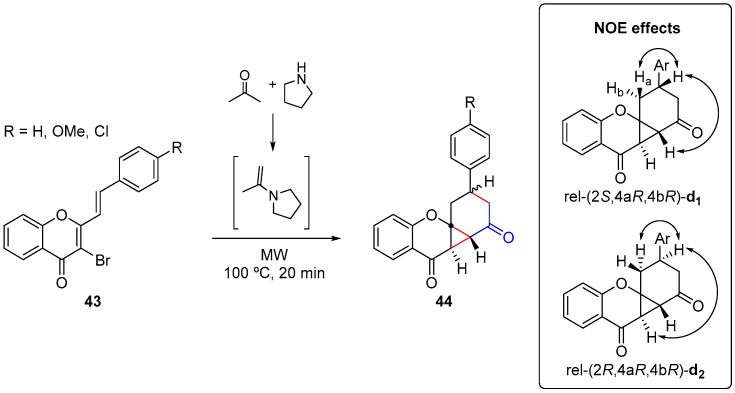
Synthesis of benzo[1,3]cyclopropa[1,2-*b*]chromene-4,5-diones **44** and NOE effects for their diastereomers using a CEM model Discover SP.

The synthesis of oxygen polycyclic benzo[1,3]cyclopropa[1,2-*b*]chromene-4,5-diones **44** involved the microwave-assisted reaction of 3-bromo-2-styryl-4*H*-chromen-4-ones **43** with 1-(prop-1-en-2-yl)pyrrolidine (generated *in situ* by the reaction of acetone with pyrrolidine) (Scheme 14) [57].

Under classical heating conditions, the polycyclic structure **44** was obtained after a 7-h reaction time. When this reaction was performed under microwave-irradiation conditions, we were able to decrease the reaction time to 20 min and, consequently, get a cleaner reaction mixture, which allowed us to fully characterize the complex diastereomeric mixture (Scheme 14, inset) of benzo[1,3]cyclopropa[1,2-*b*]chromene-4,5-diones **44** by NMR techniques [57]. The proposed mechanism for forming these fused polycyclic compounds **44** consisted of a tandem sequence of 1,6-conjugate addition, Michael-initiated ring closure, and imine hydrolysis. It is noteworthy that this cascade process was fueled by its high efficiency in producing three new C-C bonds and four new stereocenters, one of them a quaternary carbon, in one single operation.

**Scheme 15:**
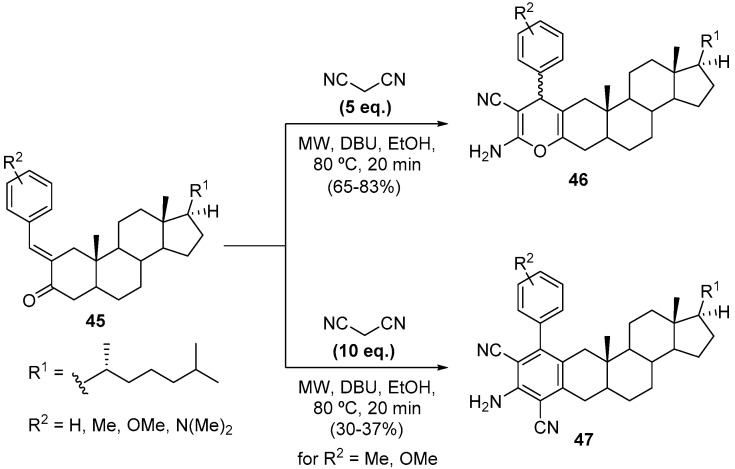
Synthesis of pyran- **46** and 2-aminoisophthalonitrile-cholestane **47**-fused compounds using a CEM model Discover SP.

Steroidal compounds are widely present in living organisms, playing important roles in their vital activities [58]. The most recognizable steroidal compound is cholesterol (cholest-5-en-3β-ol), which is considered a lipid-type molecule, being one of the most important structural components of cell membranes [59,60]. Following our ongoing interest in modified steroidal compounds for biological purposes, such as protein aggregation inhibitors, we used the MW-assisted regioselective synthesis of cholestane-fused compounds **46** and **47** (Scheme 15) [61]. In classical heating conditions, the reaction of α,β-unsaturated carbonyl substrates **45** with malononitrile is difficult to control, leading to complex reaction mixtures of several unidentified compounds. Upon application of MW irradiation, we managed to drive the reaction in different directions, carefully controlling the amount of malononitrile. This strategy allowed the efficient synthesis of pyran- **46** and 2-aminoisophthalonitrile-cholestane **47**-fused compounds, in 65–83% and 30–37% yield, respectively (Scheme 15) [61].

## 8. Miscellaneous Reactions

Among other types of reactions developed under microwave-assisted conditions, we would like to highlight the 2′-aminochalcone cyclization [17] and the oxidative aromatization [47,53,62,63] due to their importance in the synthesis of new potential bioactive heterocycles.

**Scheme 16:**
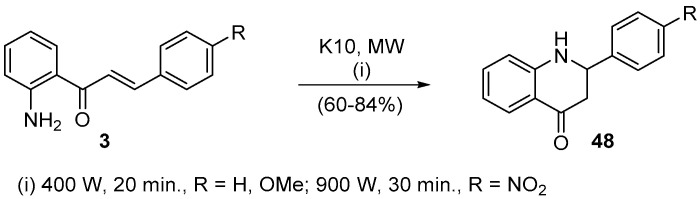
Solvent-free synthesis of azaflavanones using a Milestone-MicroSynth.

Bearing in mind that in Nature 2′-hydroxychalcones isomerize by a ring closure into flavanones, we studied the 2′-aminochalcones **3** isomerization into azaflavanones **48**, the 2-aryl-2,3-dihydroquinolin-4(1*H*)-ones (Scheme 16) [17]. Our study established a straightforward procedure using the solid support montmorillonite K10 and microwave irradiation to induce cyclization in very good yields and short reaction time. Furthermore, the desired product was obtained by removing the solid support (Scheme 16) [17].

**Scheme 17:**
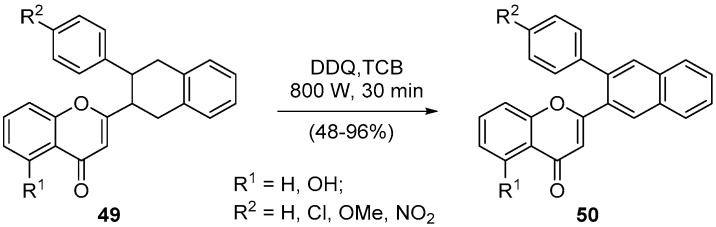
Synthesis of 2-(3-arylnaphthalen-2-yl)-4*H*-chromen-4-ones using a Milestone-MicroSynth.

Oxidative aromatization can be an important step in synthesizing new heterocyclic compounds and usually requires a long reaction time and more than one step. For example, the dehydrogenation of 2-(3-aryl-1,2,3,4-tetrahydronaphthalen-2-yl)-4*H*-chromen-4-ones **49** could be performed in a two-step procedure for 17 h, which involves the use of *N*-bromosuccinimide (NBS) [64]. Moreover, the treatment of the same 4*H*-chromen-4-ones **49** with 2,3-dichloro-5,6-dicyano-1,4-benzoquinone (DDQ) in classical thermal reflux conditions afforded some of the desired 2-(3-arylnaphthalen-2-yl)-4*H*-chromen-4-ones **50** in good yields but requires 24 h of 1,2,4-trichlorobenzene (TCB) reflux under nitrogen atmosphere [62]. The microwave irradiation assisted the aromatization, even of the 2-(3-aryl-1,2,3,4-tetrahydronaphthalen-2-yl)-4*H*-chromen-4-onse **49**-bearing electron-withdrawing groups, in better yields and in only 30-min reaction time (Scheme 17) [62]. In this example, it is evident that the microwave effect is shortening the reaction time and the accomplishment of the desired transformation for both electron-withdrawing and -donating groups.

**Scheme 18:**
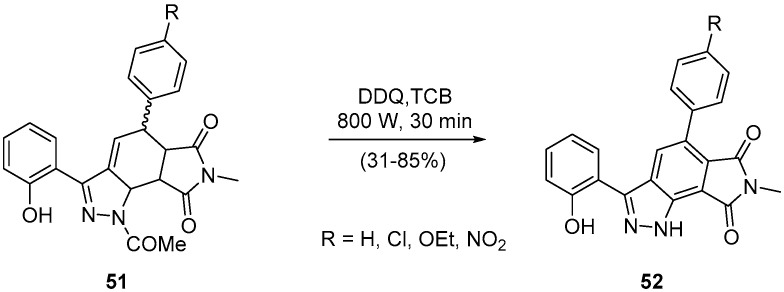
Synthesis of 5-aryl-3-(2-hydroxyphenyl)-7-methylpyrrolo[3,4-*g*]indazole-6,8(1*H*,7*H*)-diones using a Milestone-MicroSynth.

Another interesting DDQ-mediated aromatization using microwave irradiation is the synthesis of 5-aryl-3-(2-hydroxyphenyl)-7-methylpyrrolo[3,4-*g*]indazole-6,8(1*H*,7*H*)-diones **52** through the oxidation of 1-acetyl-5-aryl-3-(2-hydroxyphenyl)-7-methyl-5,5a,8a,8b-tetrahydropyrrolo[3,4-*g*]indazole-6,8(1*H*,7*H*)-diones **51**, with *N*-deacylation (Scheme 18) [53].

## 9. Conclusions

Microwave irradiation has become a widely used heating technique in organic synthesis, and our research group is no exception. Our interest in microwave-assisted synthesis has lasted almost 20 years. Over this time, our group synthesized a variety of nitrogen- and oxygen-heterocycles, resorting to this amazing heating strategy. Our experience showed us that microwave heating is a powerful tool to shorten reactions’ times from days to a few hours or even minutes. This is perhaps the main advantage that we took from using microwave irradiation. However, in a few cases, we could manage to “clean up” extremely sluggish reaction mixtures beyond the reduction of reaction time, which helped us understand complex structures and reaction mechanisms. Such features of microwaves are not often mentioned in literature reports, which mainly focus on the reduction of reaction times and improvement of the reaction yields. Another important “green” feature is that by resorting to focused microwave irradiation, it is possible to avoid using solvents, some of them highly toxic both for human health and the environment. Finally, we should not forget that microwaves also allowed us to synthesize promising dual-target compounds for Alzheimer disease, otherwise extremely difficult to obtain in appreciable amounts by classical heating conditions.

## Data Availability

Not applicable.

## References

[1] Beach E.S., Cui Z., Anastas P.T. (2009). Green Chemistry: A design framework for sustainability. Energy Environ. Sci..

[2] Mulvihill M.J., Beach E.S., Zimmerman J.B., Anastas P.T. (2011). Green Chemistry and Green Engineering: A Framework for Sustainable Technology Development. Annu. Rev. Environ. Resour..

[3] Varma R.S. (1999). Solvent-free organic syntheses using supported reagents and microwave irradiation. Green Chem..

[4] Lidström P., Tierney J., Wathey B., Westman J. (2001). Microwave assisted organic synthesis—A review. Tetrahedron.

[5] Kappe C.O. (2004). Controlled microwave heating in modern organic synthesis. Angew. Chem. Int. Ed..

[6] de la Hoz A., Díaz-Ortiz Á., Moreno A. (2005). Microwaves in organic synthesis. Thermal and non-thermal microwave effects. Chem. Soc. Rev..

[7] Díaz-Ortiz Á., Prieto P., de la Hoz A. (2019). A Critical Overview on the Effect of Microwave Irradiation in Organic Synthesis. Chem. Rec..

[8] Horikoshi S., Watanabe T., Narita A., Suzuki Y., Serpone N. (2018). The electromagnetic wave energy effect(s) in microwave–assisted organic syntheses (MAOS). Sci. Rep..

[9] Farah M., Pelle L. (2004). Microwave-assisted Chemistry in Drug Discovery. Curr. Topics Med. Chem..

[10] Reis J., Gaspar A., Milhazes N., Borges F. (2017). Chromone as a Privileged Scaffold in Drug Discovery: Recent Advances. J. Med. Chem..

[11] Dhiman P., Arora N., Thanikachalam P.V., Monga V. (2019). Recent advances in the synthetic and medicinal perspective of quinolones: A review. Bioorg. Chem..

[12] Li J.J. (2009). Aldol condensation. Name Reactions: A Collection of Detailed Mechanisms and Synthetic Applications.

[13] Nielsen A.T., Houlihan W.J. (2011). The Aldol Condensation. Organic Reactions.

[14] Rozmer Z., Perjési P. (2016). Naturally occurring chalcones and their biological activities. Phytochem. Rev..

[15] Silva A.M.S., Tavares H.R., Barros A.I.N.R.A., Cavaleiro J.A.S. (1997). NMR and Structural and Conformational Features of 2′-Hydroxychalcones and Flavones. Spectrosc. Lett..

[16] Barros A.I.R.N.A., Silva A.M.S. (2006). Efficient Synthesis of Nitroflavones by Cyclodehydrogenation of 2′-Hydroxychalcones and by the Baker-Venkataraman Method. Monatsh. Chem..

[17] Rocha D.H.A., Vaz P.A.A.M., Pinto D.C.G.A., Silva A.M.S. (2019). Synthesis Chalones and Their Isomerization into Flavanones and Azaflavanones. Methods Protoc..

[18] Silva C.F.M., Batista V.F., Pinto D.C.G.A., Silva A.M.S. (2018). Challenges with chromone as a privileged scaffold in drug discovery. Expert Opin. Drug Discov..

[19] Baker W. (1933). Molecular rearrangement of some o-acyloxyacetophenones and the mechanism of the production of 3-acylchromones. J. Chem. Soc..

[20] Pinto D.C.G.A., Silva A.M.S., Cavaleiro J.A.S. (2000). A convenient synthesis of new (*E*)-5-hydroxy-2-styrylchromones by modifications of the Baker–Venkataraman method. New J. Chem..

[21] Pinto D.C.G.A., Silva A.M.S., Almeida L.M.P.M., Cavaleiro J.A.S., Elguero J. (2002). 3-Aroyl-5-hydroxyflavones: Synthesis and Transformation into Aroylpyrazoles. Eur. J. Org. Chem..

[22] Pinto D.C.G.A., Silva A.M.S., Cavaleiro J.A.S. (2007). Baker-Venkataraman Rearrangement Under Microwave Irradiation: A New Strategy for the Synthesis of 3-Aroyl-5-hydroxyflavones. Synlett.

[23] Pinto D.C.G.A., Seca A.M.L., Leal S.B., Silva A.M.S., Cavaleiro J.A.S. (2011). A Novel Short-Step Synthesis of New Xanthenedione Derivatives from the Cyclization of 3-Cinnamoyl-2-styrylchromones. Synlett.

[24] Almeida A.I.S., Silva V.L.M., Silva A.M.S., Pinto D.C.G.A., Cavaleiro J.A.S. (2008). Syntheses of Novel (*E*)-N-Methyl-2-styryl-4-quinolones. Synlett.

[25] Knoevenagel E. (1922). Obituary. Angew. Chem..

[26] Knoevenagel E. (1898). Condensation von Malonsäure mit aromatischen Aldehyden durch Ammoniak und Amine. Ber. Dtsch. Chem. Ges..

[27] Knoevenagel E. (1903). Ueber Condensationsproducte von Acetylaceton mit Aldehyden. Ber. Dtsch. Chem. Ges..

[28] Heravi M.M., Janati F., Zadsirjan V. (2020). Applications of Knoevenagel condensation reaction in the total synthesis of natural products. Monatsh. Chem..

[29] Silva V.L.M., Silva A.M.S., Pinto D.C.G.A., Cavaleiro J.A.S., Patonay T. (2004). Condensation of Chromone-3-carboxaldehyde with Phenylacetic Acids: An Efficient Synthesis of (*E*)-3-Styrylchromones. Synlett.

[30] Silva V.L.M., Silva A.M.S., Pinto D.C.G.A., Cavaleiro J.A.S., Vasas A., Patonay T. (2008). Syntheses of (*E*)- and (*Z*)-3-styrylchromones. Monatsh. Chem..

[31] Patonay T., Kiss-Szikszai A., Silva V.M.L., Silva A.M.S., Pinto D.C.G.A., Cavaleiro J.A.S., Jekő J. (2008). Microwave-Induced Synthesis and Regio- and Stereoselective Epoxidation of 3-Styrylchromones. Eur. J. Org. Chem..

[32] Heck R.F., Nolley J.P. (1972). Palladium-catalyzed vinylic hydrogen substitution reactions with aryl, benzyl, and styryl halides. J. Org. Chem..

[33] Heck R.F. (1982). Palladium-Catalyzed Vinylation of Organic Halides. Organic Reactions.

[34] Mc Cartney D., Guiry P.J. (2011). The asymmetric Heck and related reactions. Chem. Soc. Rev..

[35] Dounay A.B., Overman L.E. (2003). The Asymmetric Intramolecular Heck Reaction in Natural Product Total Synthesis. Chem. Rev..

[36] Reznikov A.N., Ashatkina M.A., Klimochkin Y.N. (2021). Recent developments in asymmetric Heck type cyclization reactions for constructions of complex molecules. Org. Biomol. Chem..

[37] Singh B.K., Kaval N., Tomar S., Eycken E.V.D., Parmar V.S. (2008). Transition Metal-Catalyzed Carbon−Carbon Bond Formation Suzuki, Heck, and Sonogashira Reactions Using Microwave and Microtechnology. Org. Process. Res. Dev..

[38] Rocha D.H.A., Pinto D.C.G.A., Silva A.M.S., Patonay T., Cavaleiro J.A.S. (2012). A New Synthesis of 5-Arylbenzo[c]xanthones from Photoinduced Electrocyclisation and Oxidation of (*E*)-3-Styrylflavones. Synlett.

[39] Rocha D.H.A., Pinto D.C.G.A., Silva A.M.S. (2015). Synthesis and cyclisation studies of (*E*)-2-aryl-1-methyl-3-styrylquinolin-4(1H)-ones. Tetrahedron.

[40] Diels O., Alder K. (1928). Synthesen in der hydroaromatischen Reihe. Liebigs Ann. Chem..

[41] Berson J.A. (1992). Discoveries missed, discoveries made: Creativity, influence, and fame in chemistry. Tetrahedron.

[42] Nicolaou K.C., Snyder S.A., Montagnon T., Vassilikogiannakis G. (2002). The Diels–Alder Reaction in Total Synthesis. Angew. Chem. Int. Ed..

[43] Tasdelen M.A. (2011). Diels–Alder “click” reactions: Recent applications in polymer and material science. Polym. Chem..

[44] Gregoritza M., Brandl F.P. (2015). The Diels–Alder reaction: A powerful tool for the design of drug delivery systems and biomaterials. Eur. J. Pharm. Biopharm..

[45] Pinto D.C.G.A., Silva A.M.S., Almeida L.M.P.M., Carrillo J.R., Díaz-Ortiz A., de la Hoz A., Cavaleiro J.A.S. (2003). First Diels-Alder Reactions of 3-Styrylchromones under Microwave Irradiation. Synlett.

[46] Sandulache A., Silva A.M.S., Pinto D.C.G.A., Almeida L.M.P.M., Cavaleiro J.A.S. (2003). Wittig reactions of chromone-3-carboxaldehydes with benzylidenetriphenyl phosphoranes: A new synthesis of 3-styrylchromones. New J. Chem..

[47] Pinto D.C.G.A., Silva A.M.S., Brito C.M., Sandulache A., Carrillo J.R., Prieto P., Díaz-Ortiz A., de la Hoz A., Cavaleiro J.A.S. (2005). Reactivity of 3-Styrylchromones as Dienes in Diels–Alder Reactions under Microwave Irradiation: A New Synthesis of Xanthones. Eur. J. Org. Chem..

[48] Albuquerque H.M.T., Santos C.M.M., Cavaleiro J.A.S., Silva A.M.S. (2015). (*E*)-2-(4-Arylbut-1-en-3-yn-1-yl)chromones as Synthons for the Synthesis of Xanthone-1,2,3-triazole Dyads. Eur. J. Org. Chem..

[49] Albuquerque H.M.T., Santos C.M.M., Lima C.F.R.A.C., Santos L.M.N.B.F., Cavaleiro J.A.S., Silva A.M.S. (2017). 2-[(1*E*,3*E*)-4-Arylbuta-1,3-dien-1-yl]-4H-chromen-4-ones as Dienes in Diels–Alder Reactions—Experimental and Computational Studies. Eur. J. Org. Chem..

[50] Almeida A.I.S., Silva V.L.M., Silva A.M.S., Pinto D.C.G.A., Cavaleiro J.A.S. (2012). Diels-Alder Reactions of (*E*)-2-Styrylquinolin-4(1H)-ones with N-Methylmaleimide: New Syntheses of Acridin-9(10H)-ones. Synlett.

[51] Silva V.L.M., Silva A.M.S., Pinto D.C.G.A., Cavaleiro J.A.S., Elguero J. (2007). Novel (*E*)- and (*Z*)-3(5)-(2-hydroxyphenyl)-4-styrylpyrazoles from (*E*)- and (*Z*)-3-styrylchromones: The unexpected case of (*E*)-3(5)-(2-hydroxyphenyl)-4-(4-nitrostyryl)pyrazoles. Tetrahedron Lett..

[52] Silva V.L.M., Silva A.M.S., Pinto D.C.G.A., Cavaleiro J.A.S., Elguero J. (2008). Synthesis of (*E*)- and (*Z*)-3(5)-(2-hydroxyphenyl)-4-styrylpyrazoles. Monatsh. Chem..

[53] Silva V.L.M., Silva A.M.S., Pinto D.C.G.A., Cavaleiro J.A.S. (2006). Efficient Microwave-Assisted Synthesis of Tetrahydroindazoles and their Oxidation to Indazoles. Synlett.

[54] Silva V.L.M., Silva A.M.S., Pinto D.C.G.A., Elguero J., Cavaleiro J.A.S. (2009). Synthesis of New 1H-Indazoles through Diels–Alder Transformations of 4-Styrylpyrazoles under Microwave Irradiation Conditions. Eur. J. Org. Chem..

[55] Albuquerque H.M.T., Santos C.M.M., Cavaleiro J.A.S., Silva A.M.S. (2018). First intramolecular Diels–Alder reactions using chromone derivatives: Synthesis of chromeno[3,4-b]xanthones and 2-(benzo[c]chromenyl)chromones. New J. Chem..

[56] Malafaia D., Oliveira A., Fernandes P.A., Ramos M.J., Albuquerque H.M.T., Silva A.M.S. (2021). Chromeno[3,4-b]xanthones as First-in-Class AChE and Aβ Aggregation Dual-Inhibitors. Int. J. Mol. Sci..

[57] Sousa J.L.C., Silva A.M.S. (2017). Microwave-Assisted Tandem Reactions towards a New Synthesis of Cyclohepta[b]chromene-9,11-diones. Synlett.

[58] Albuquerque H.M.T., Santos C.M.M., Silva A.M.S. (2019). Cholesterol-Based Compounds: Recent Advances in Synthesis and Applications. Molecules.

[59] Nes W.D. (2011). Biosynthesis of Cholesterol and Other Sterols. Chem. Rev..

[60] Cerqueira N.M., Oliveira E.F., Gesto D.S., Santos-Martins D., Moreira C., Moorthy H.N., Ramos M.J., Fernandes P.A. (2016). Cholesterol Biosynthesis: A Mechanistic Overview. Biochemistry.

[61] Albuquerque H.M.T., Francisco T., Malafaia D., Cavaleiro J.A.S., Silva A.M.S. (2021). A-ring functionalization of cholestane with highly substituted pyrans and 2-aminoisophthalonitriles. Arkivoc.

[62] Patoilo D.T., Silva A.M.S., Pinto D.C.G.A., Tomé A.C., Cavaleiro J.A.S. (2007). Synthesis of 5-Hydroxy-2-(naphth-2-yl)chromone derivatives. J. Heterocycl. Chem..

[63] Seca A.M.L., Leal S.B., Pinto D.C.G.A., Barreto M.C., Silva A.M.S. (2014). Xanthenedione Derivatives, New Promising Antioxidant and Acetylcholinesterase Inhibitor Agents. Molecules.

[64] Silva A.M.S., Silva A.M.G., Tomé A.C., Cavaleiro J.A.S. (1999). New Syntheses of Flavones from Diels–Alder Reactions of 2-Styrylchromones with ortho-Benzoquinodimethanes. Eur. J. Org. Chem..

